# Supplementary material for: Integrated analysis of telomerase enzymatic activity unravels an association with cancer stemness and proliferation
Source: Nat Commun. 2021 Jan 8;12:139. doi: 10.1038/s41467-020-20474-9 (PMC7794223; doi:10.1038/s41467-020-20474-9)
Supplement: Supplementary file 1 — Supplementary Information [file 41467_2020_20474_MOESM1_ESM.pdf]

**Supplementary Fig. 1. (a)** Schematic of EXpression-based Telomerase Enzymatic activity Detection (EXTEND) tool workflow. GE, Gene Expression; LGG, lower grade glioma (from TCGA). On the right is the schematic of the computing procedure.

a

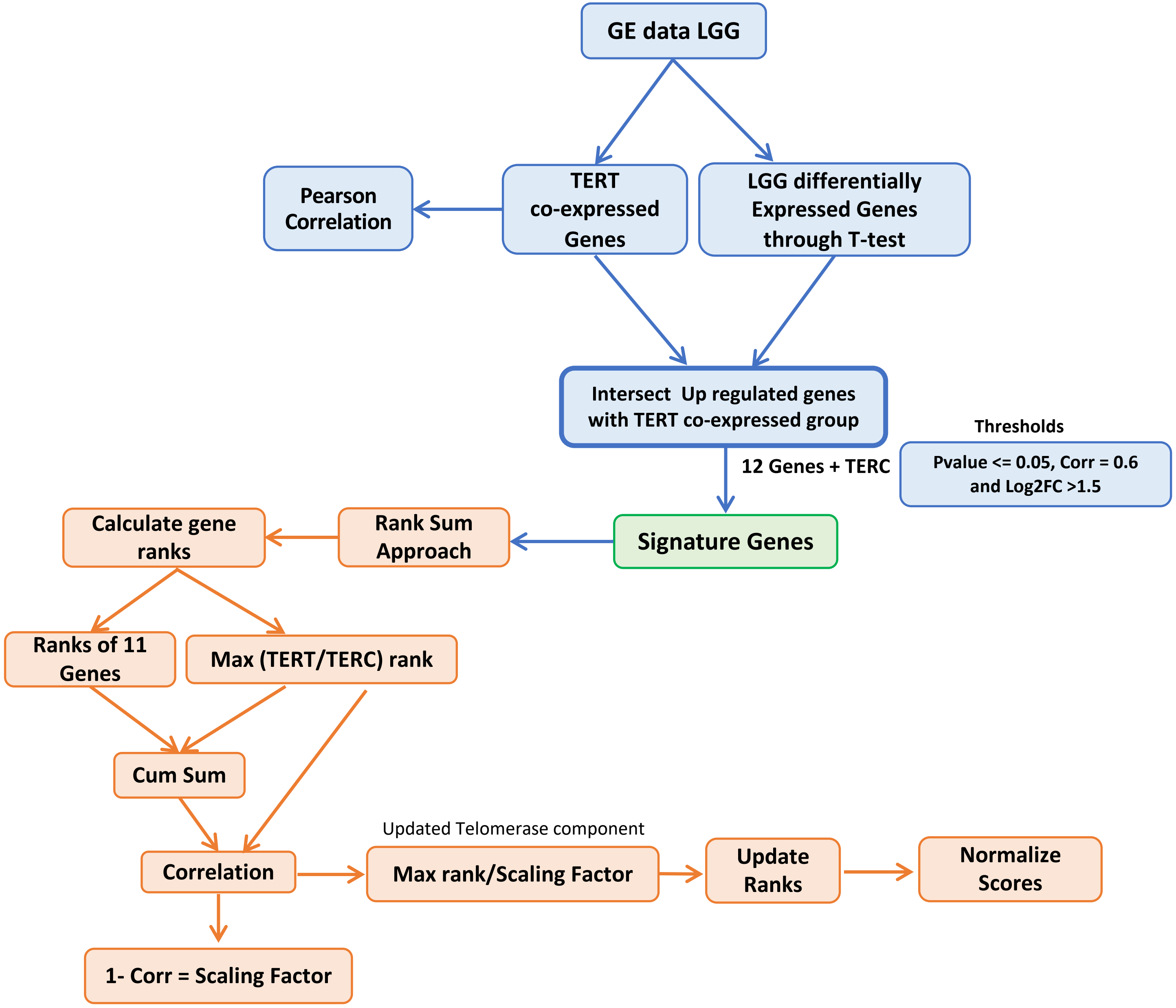

**Supplementary Fig. 1. (b)** Schematic of EXTEND computing procedure.

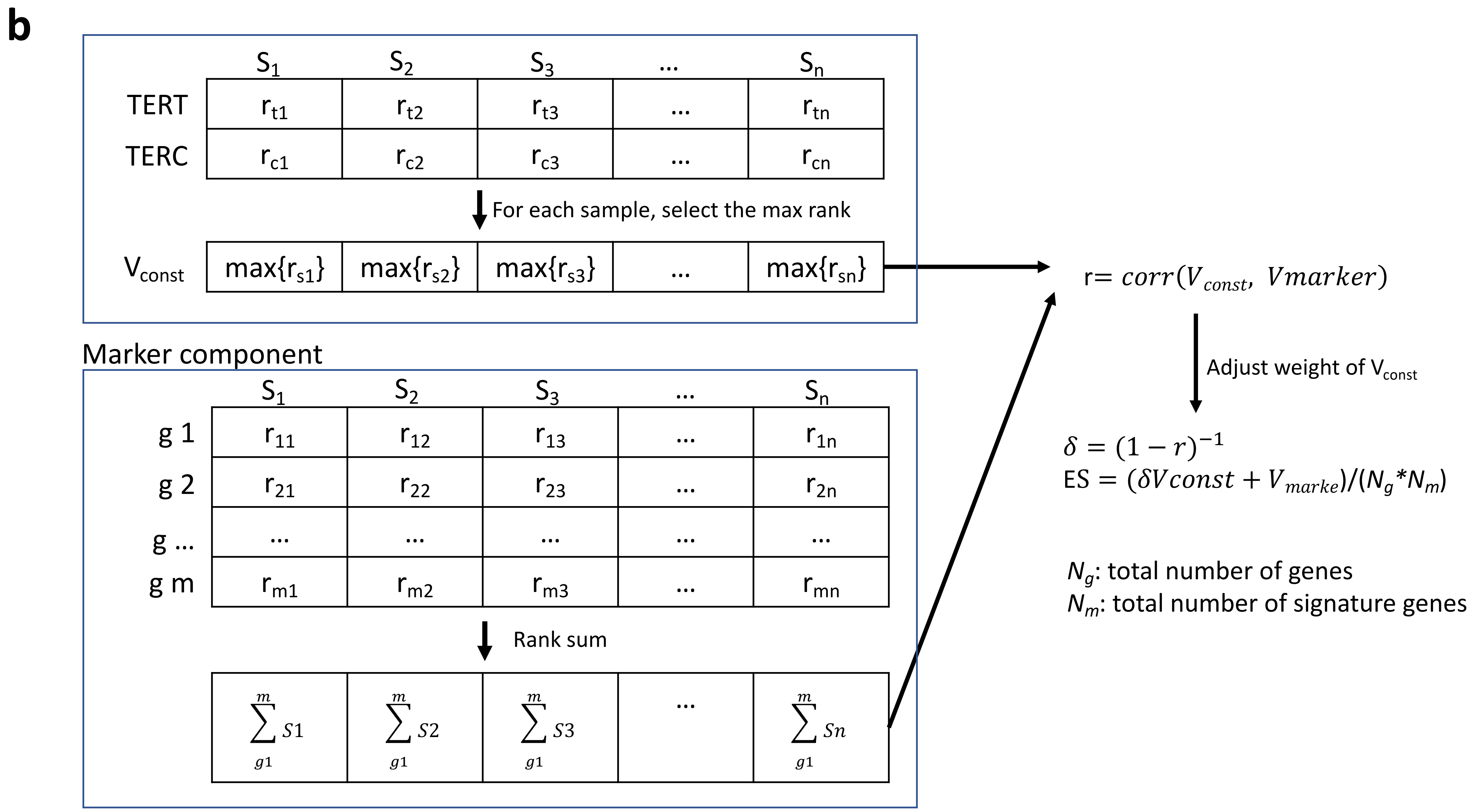

**Supplementary Fig. 2.** (**a**) Frequencies of *ATRX*/*DAXX* alteration and *TERT* promoter mutation across TCGA cancer cohorts. X-axis indicates sample size. Each bar represents one cancer type from TCGA. (**b**) *TERT* promoter mutation and *ATRX* mutations are strongly mutually exclusive in LGG. Data used from Barthel et al.,2017.

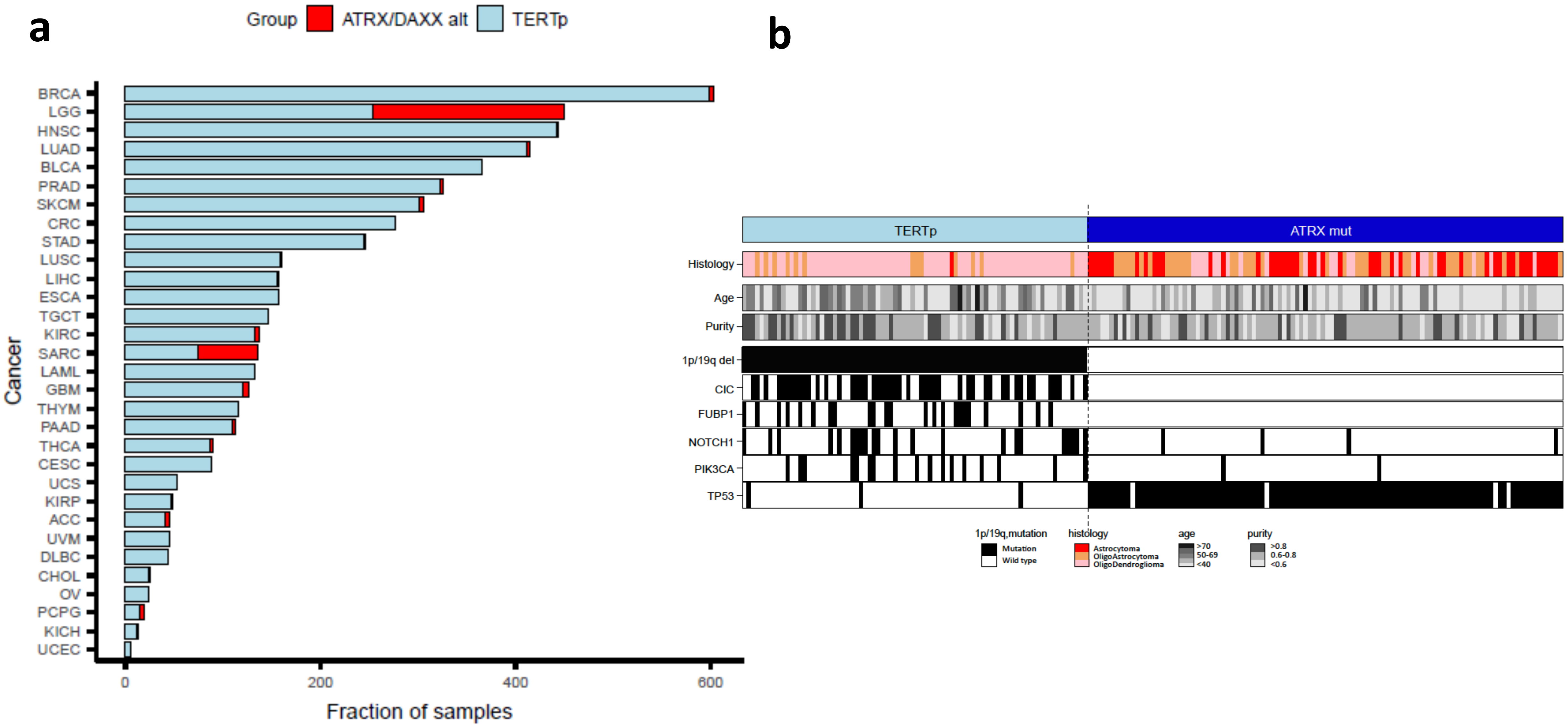

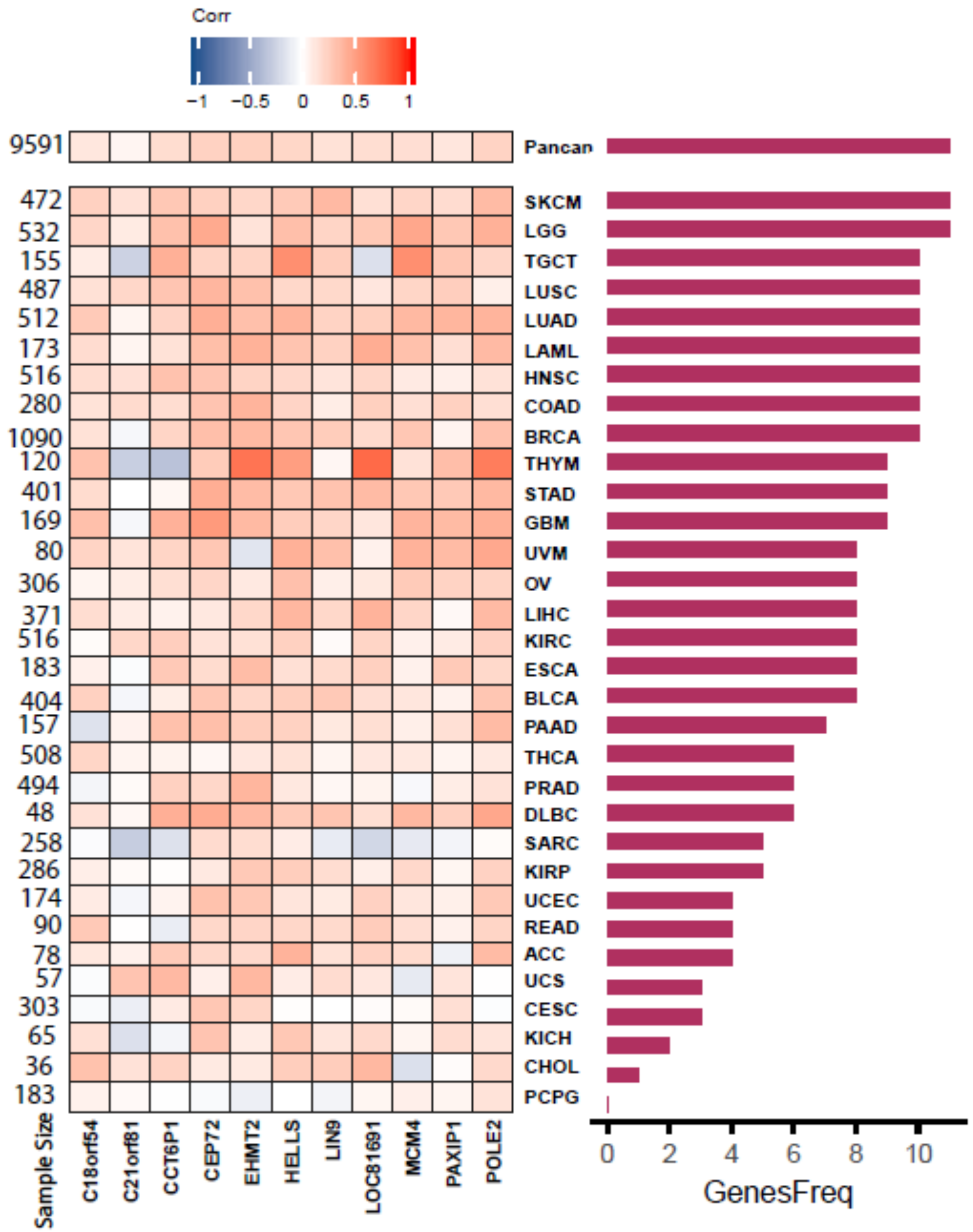


**Supplementary Fig. 3.** Correlation of *TERT* expression with remaining 11 signature genes across 32 cancer types in TCGA. Color intensity of heatmap represents Pearson correlation coefficient, (red = positive and blue = negative) while the bar plot on right hand side represents number of genes passing p-value threshold of 0.05. Numbers on the left denote sample size. Data used is available in Source Data.

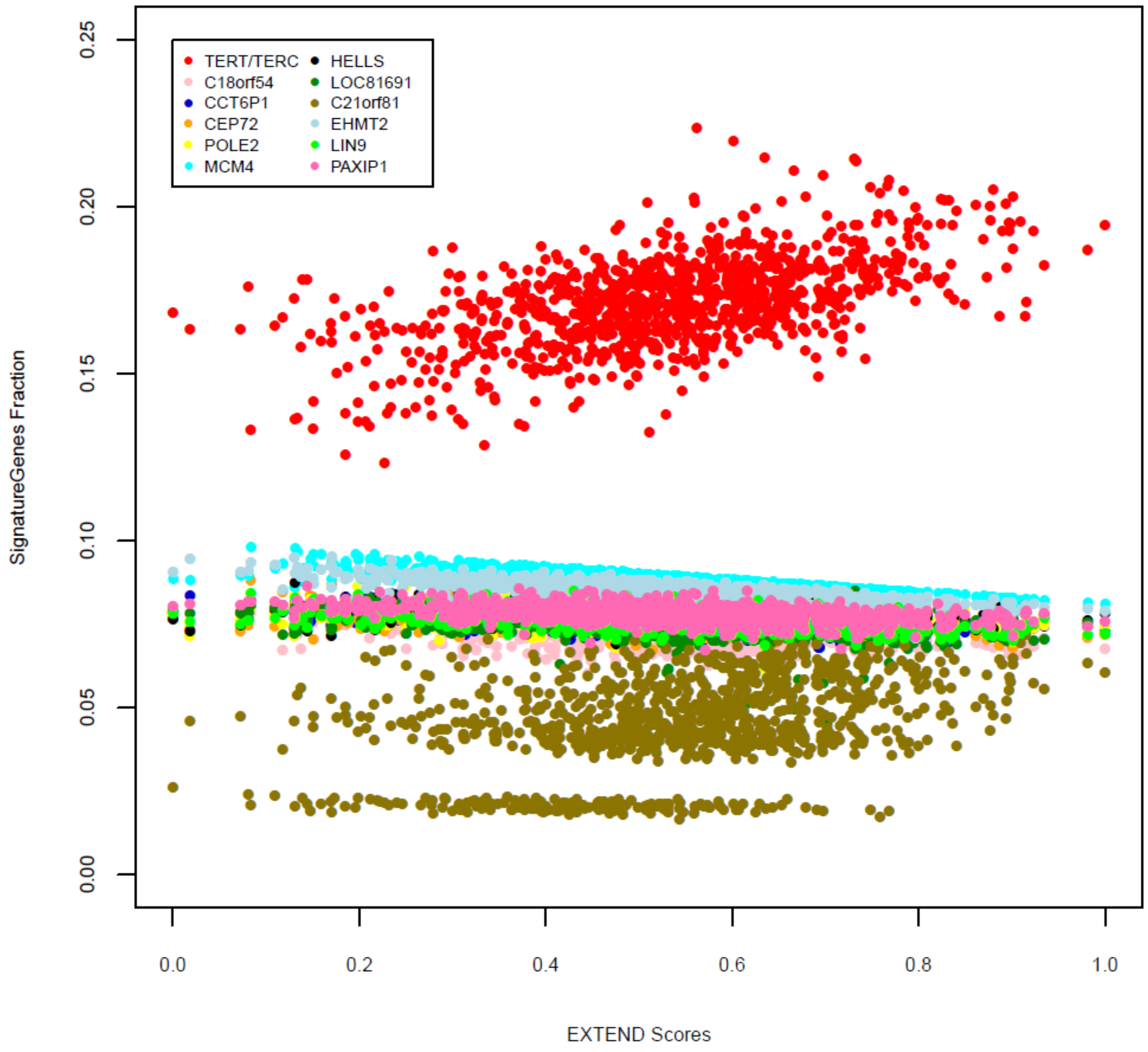


**Supplementary Fig. 4.** Contribution of signature genes towards EXTEND score evaluated using Cancer Cell Line Encyclopedia (CCLE) data. Each dot represents one cell line. *TERT/TERC* contributes more than other genes in the signature but overall the contribution is less than 20%. Data used is available in Source Data.

**Supplementary Fig. 5.** Spearman correlation between PolyA enrichment protocol-based prediction and ribosomal depletion protocol-based prediction. Data from GSE51783 was used for this analysis. X-axis represents PolyA enrichment protocol-based scores, whereas y-axis represents ribosomal depletion protocol-based scores. The bars are the connecting lines for samples with their respective labels. Shade indicates 95% CI of the regression. Data used is available in Source Data.

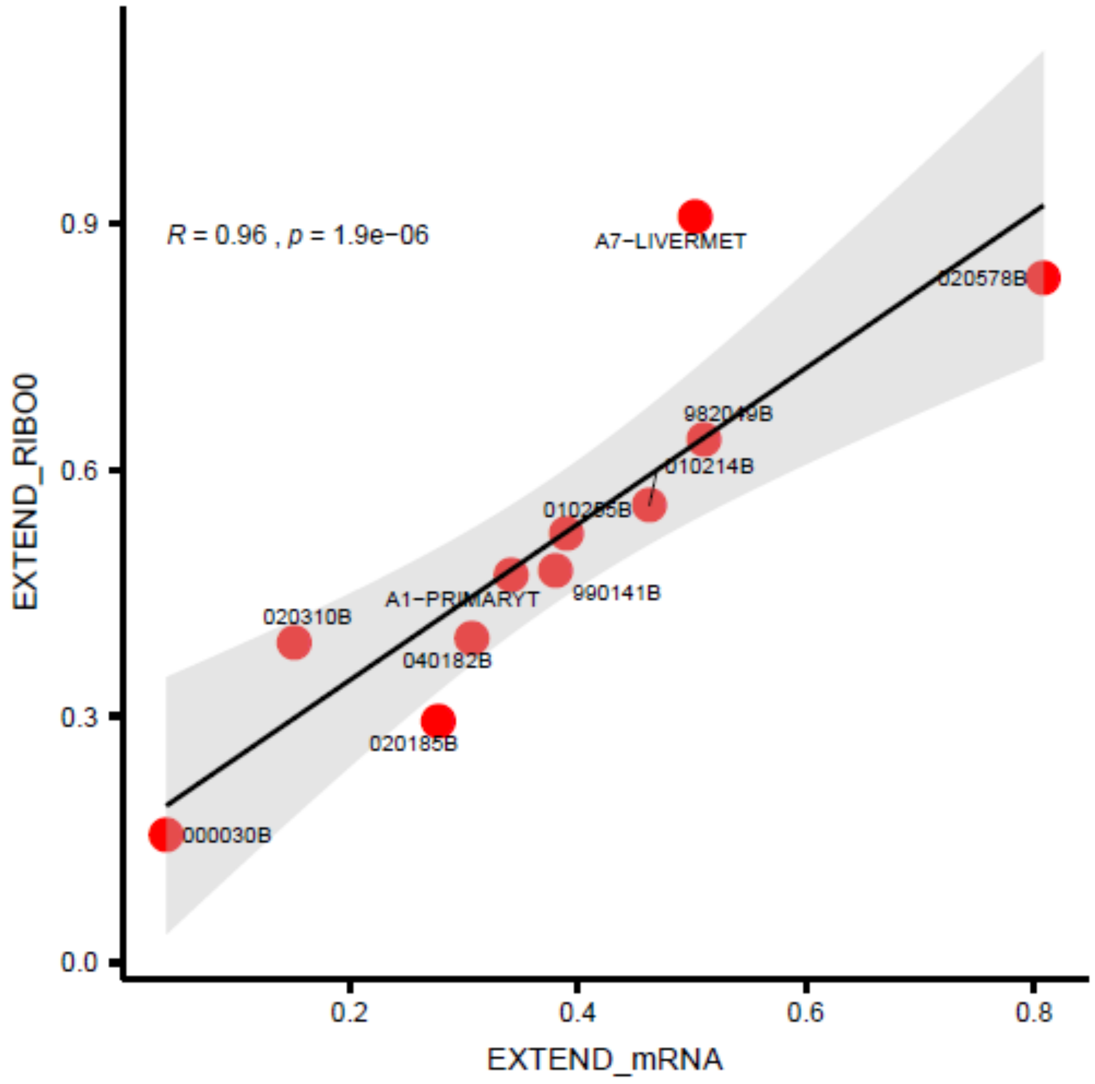

**Supplementary Fig. 6.** Spearman correlation of telomerase activity (measured by TRAP assay) with **(a)** EXTEND scores (Fig. 1a) and **(b)** *TERT* expression across 28 glioma cell lines. Each dot represents a cell line. X-axis in both panels denotes telomerase activity from TRAP assays. Y-axis is EXTEND score and *TERT* expression, respectively. GSC5-22 and GSC818 are two ALT lines. The bars are the connecting lines for cell lines with their respective labels. Shade indicates 95% CI of the regression. Data used is available in Source Data.

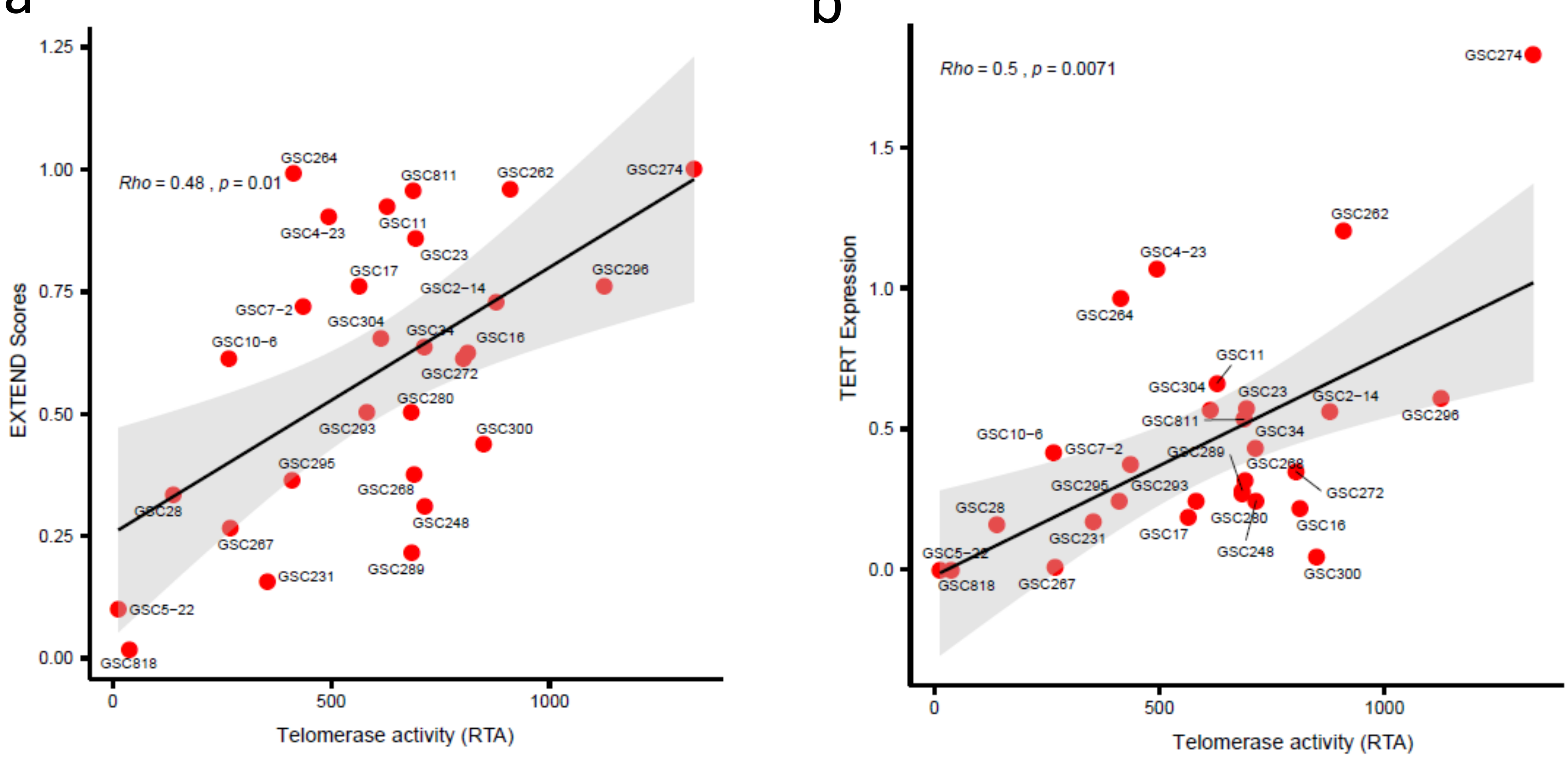

**Supplementary Fig. 7.** Spearman correlation of telomerase activity (obtained through enzymatic assay) with **(a)** EXTEND scores and **(b)** *TERT* expression across 11 bladder cancer cell lines. Correlation coefficient (Rho = 0.72, P-value = 0.013) of EXTEND scores versus telomerase activity significantly outperforms *TERT* expression (Rho= 0.55, P-value = 0.083). Shade indicates 95% CI of the regression. Data used is available in Source Data.

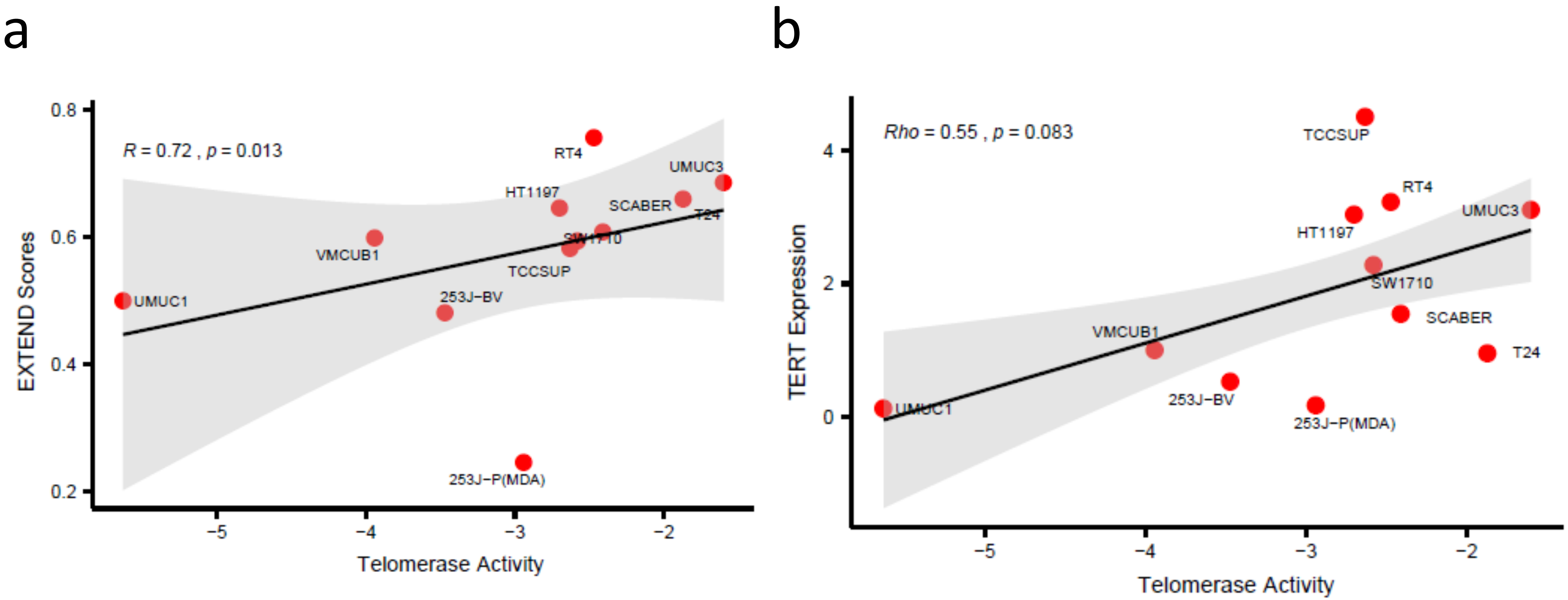

**Supplementary Fig. 8**. Spearman correlation of telomerase activity (obtained through digital droplet-based TRAP assay) with **(a)** EXTEND scores and **(b)** *TERT* expression across 15 lung cancer cell lines. Correlation coefficient (Rho = 0.65, P-value = 0.01) of EXTEND scores versus telomerase activity significantly outperforms *TERT* expression (Rho= 0.48, P-value = 0.07). The bars are the connecting lines for cell lines with their respective labels. Shade indicates 95% CI of the regression. Data used is available in Source Data.

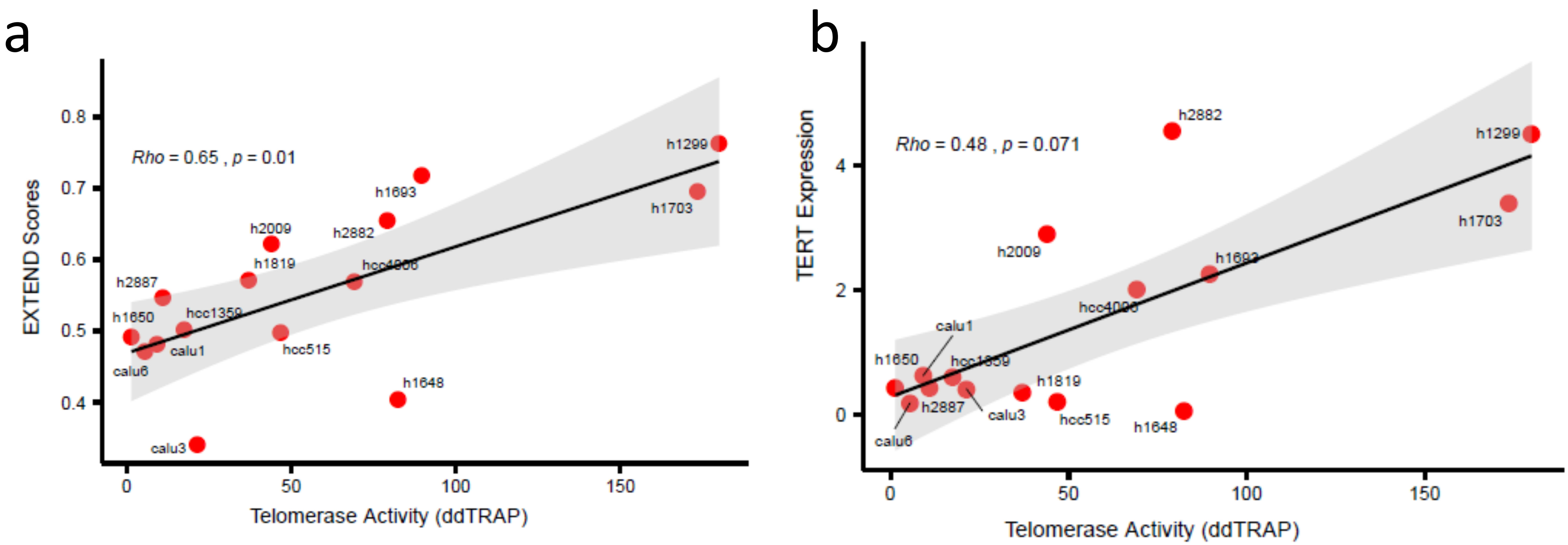

**Supplementary Fig. 9**. Comparison of (**a**) *TERT* expression and (**b**) EXTEND Scores in liposarcomas ALT (n = 10) and telomerase positive (n =8) tumors (GSE14533). Both telomerase positive tumors and cell lines (n = 8) show significantly higher EXTEND scores and TERT expression than ALT samples (n = 8; cell lines). EXTEND achieves a better performance (Two sided t-test P-value 7e-6 vs 0.003 for cell lines, 0.001 vs 0.009 for tumor samples). Box plots, interquartile ranges (25th to 75th percentile); middle bar defines median and the minima and maxima are within 1.5 times the interquartile range of the lower and higher quartile. Data used is available in Source Data.

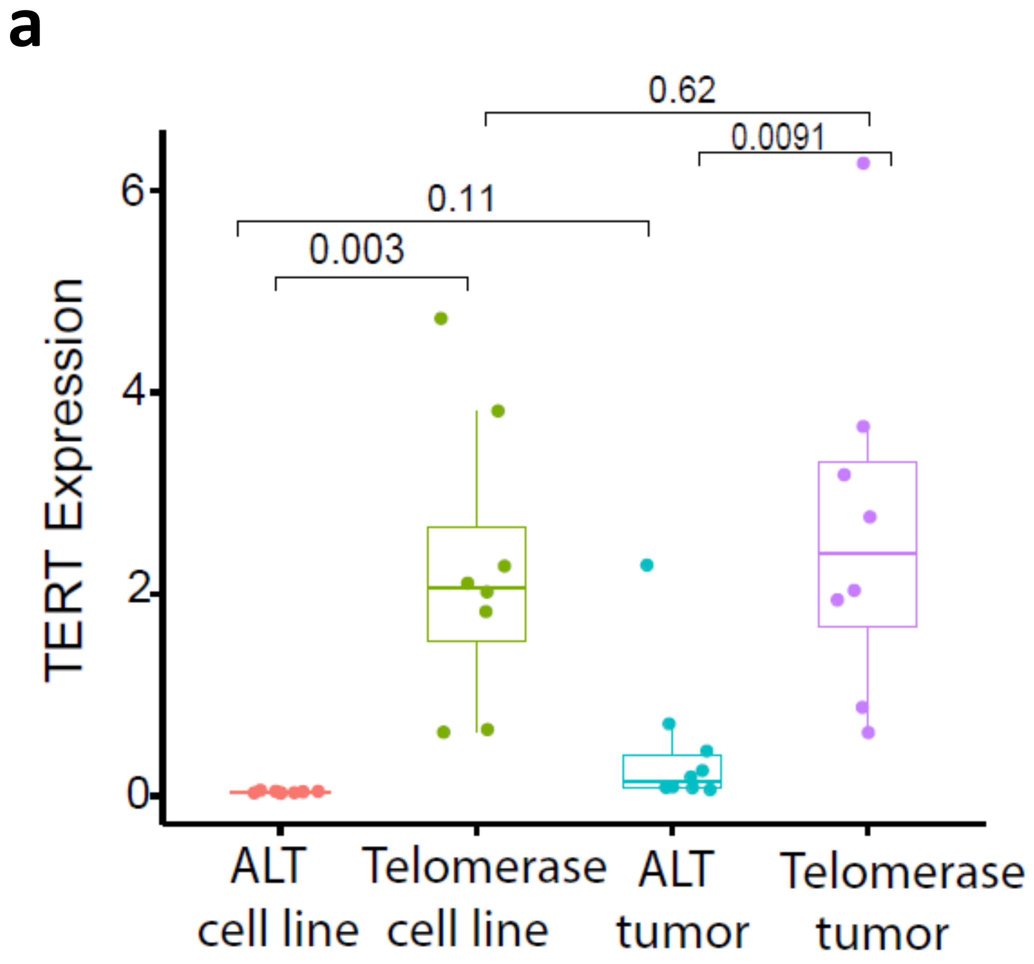


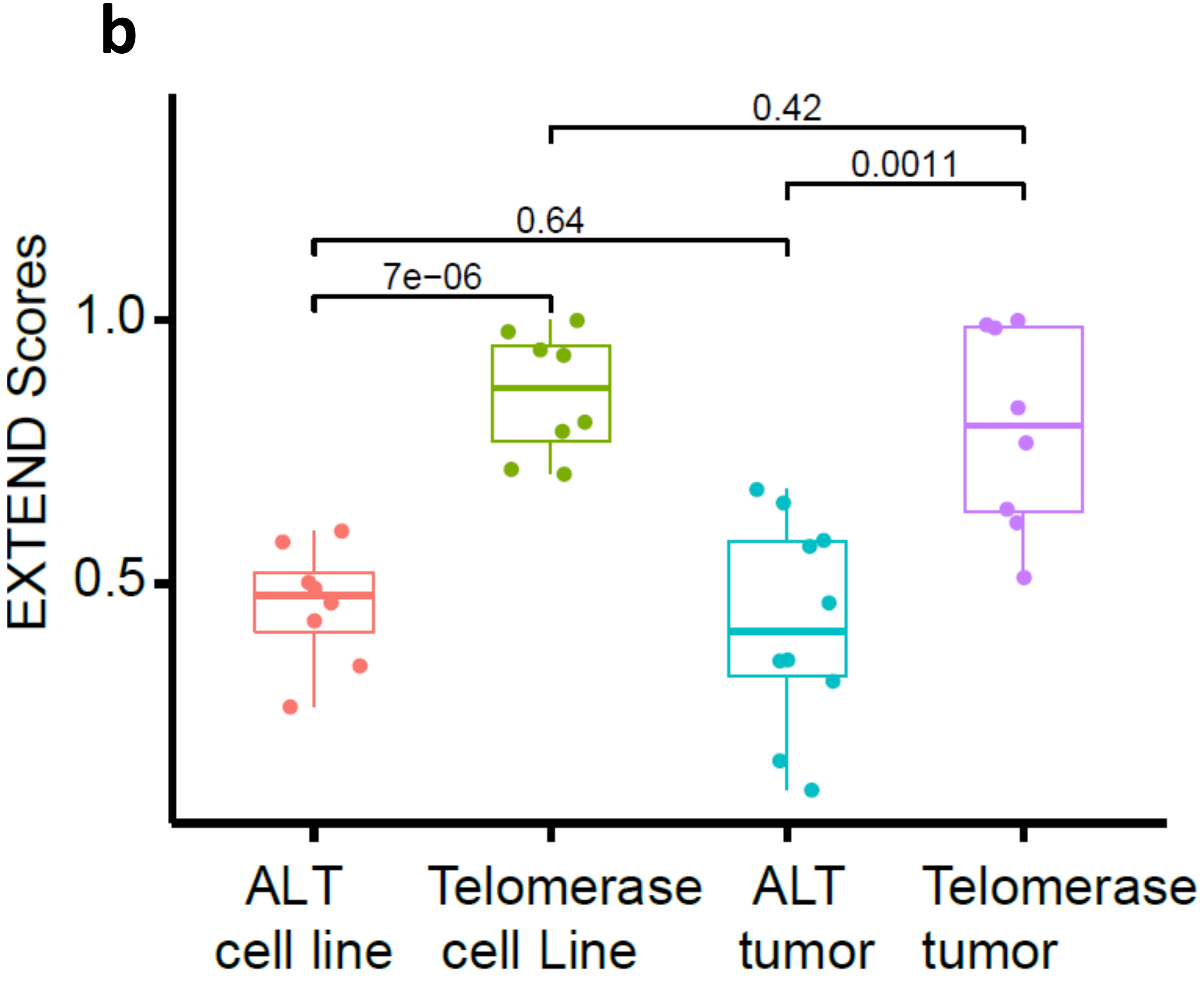

**Supplementary Fig. 10.** Comparison of **(a)** *TERT* expression and (b) EXTEND scores across the five TMM groups of Neuroblastoma (GSE120572). Telomerase positive groups (*MYCN* amplification (n = 52), *TERT* high (n = 9) and *TERT* rearrangement (n = 21) show significantly higher scores than ALT (n = 31) and no-TMM (n = 99) groups). EXTEND pattern for telomerase positive groups is same as experimental data shown in Ackerman et al., 2018 study. Box plots, interquartile ranges (25th to 75th percentile); middle bar defines median and the minima and maxima are within 1.5 times the interquartile range of the lower and higher quartile. Statistical differences were assessed using two-sided student's t-test. Data used is available in Source Data.

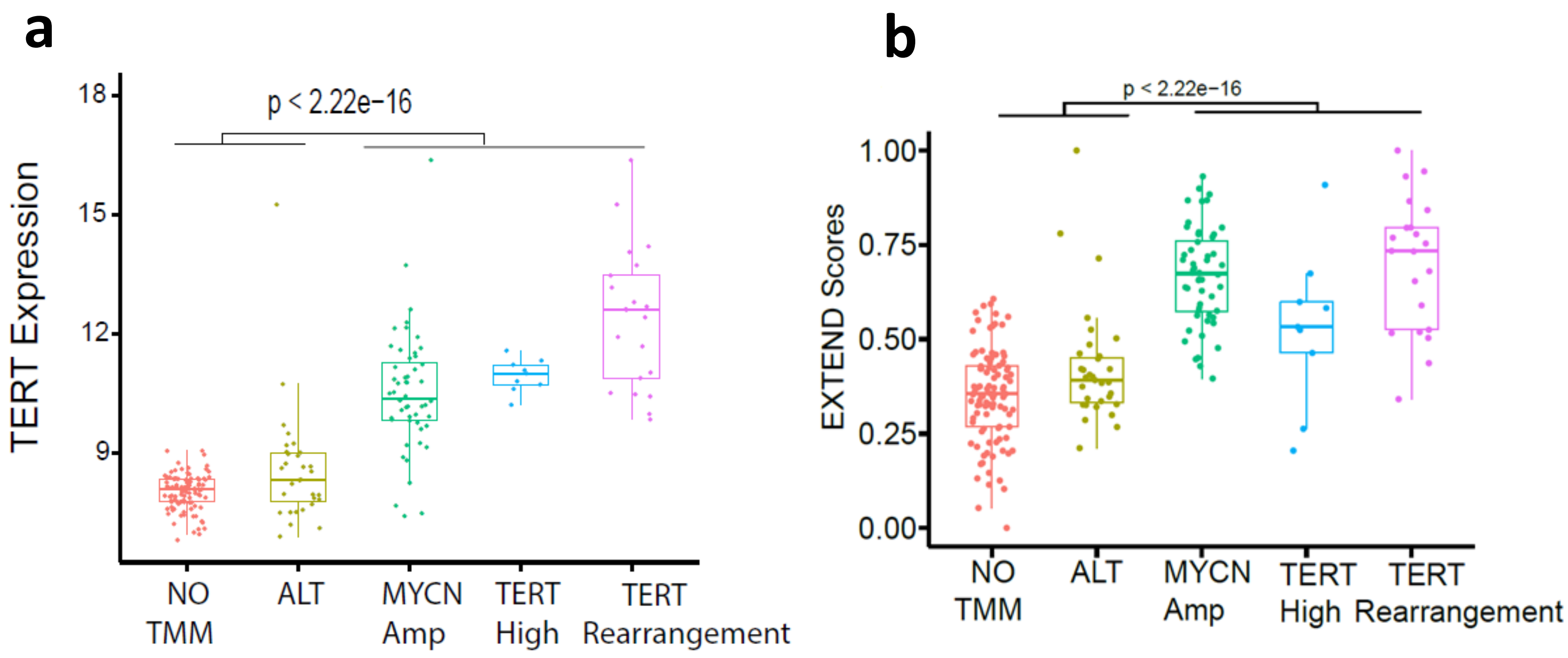

**Supplementary Fig. 11.** Distribution of EXTEND scores across human tissues (Total cases, n = 11688). Each group represents one tissue type from GTEx. *TERT* expression is denoted by the left y axis (sea green), whereas EXTEND score (pink) is denoted by the right y axis. Boxplot interquartile ranges ($25^{th}$ to $75^{th}$ percentile); middle bar defines median and the minima and maxima are within 1.5 times the interquartile range of the lower and higher quartile. Data used is available in Source Data.

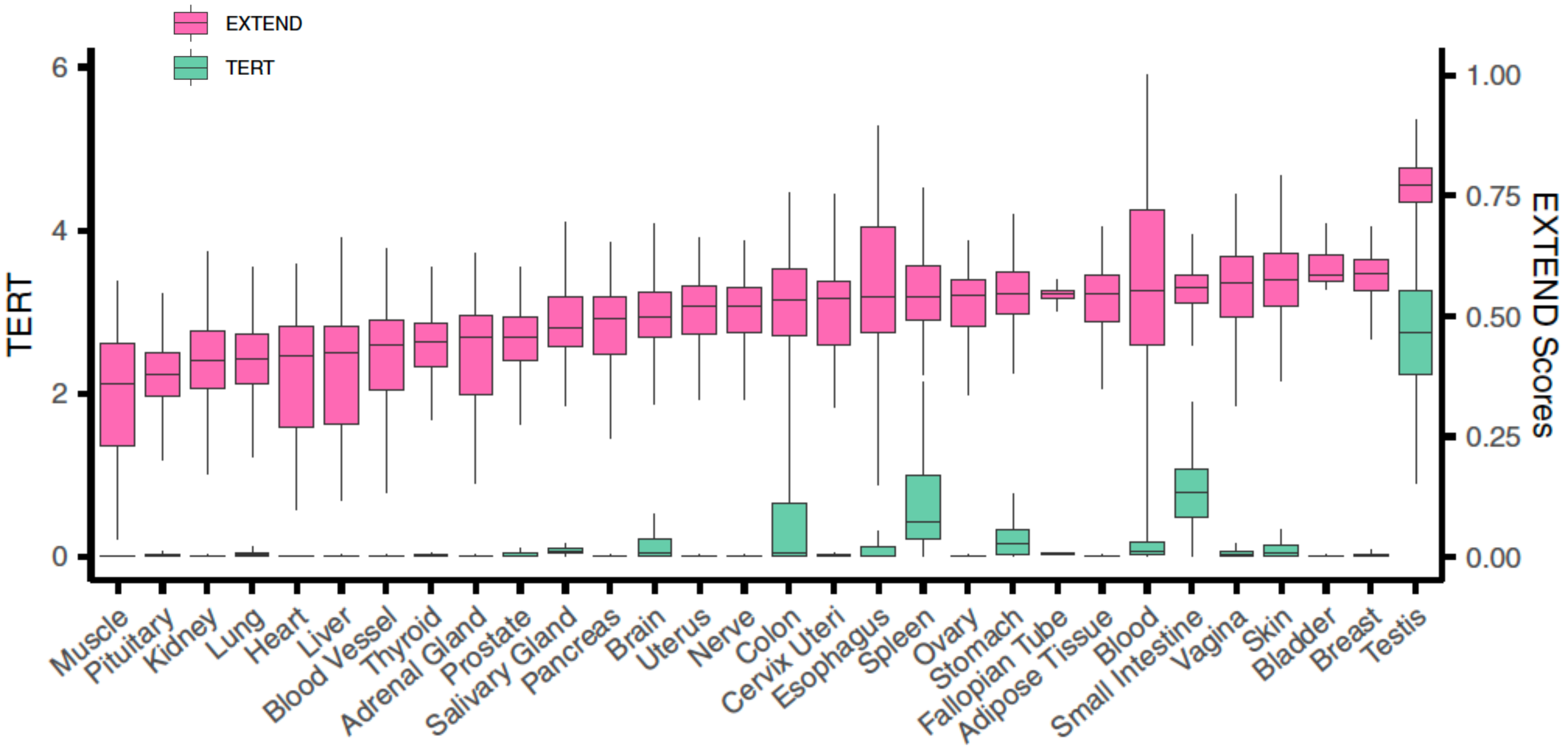

**Supplementary Fig. 12.** A screenshot from UCSC Xena shows the percentages of *TERT* isoforms in brain (purple) versus testis (cyan) across GTEx samples. Most of *TERT* expressed in the brain are short spliced forms (second last), whereas the proportion of full-length isoform is close to zero. In contrast, about 25-30% of *TERT* expressed in the testis are full-length isoforms.

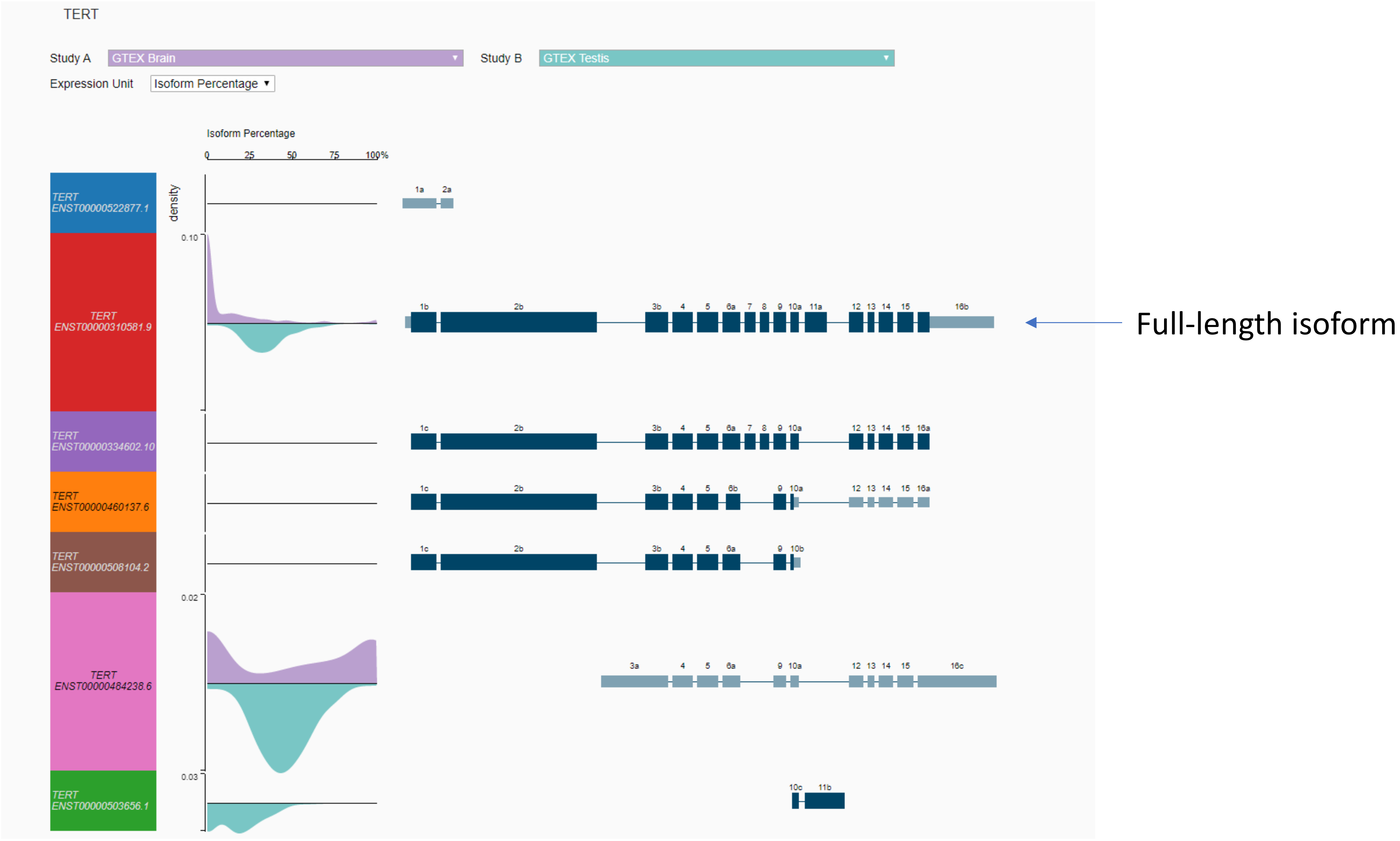

**Supplementary Fig. 13.** Spearman correlation between EXTEND and *POT1* across TCGA pan-cancer (left). No correlation was observed between *POT1* and *TERT* expression (right). For this analysis, EXTEND scores and gene expression were z-score transformed in each cancer cohort to remove tissue effect.  Each dot reflect one sample (n equivalent to 9000). Data used is available in Source Data.

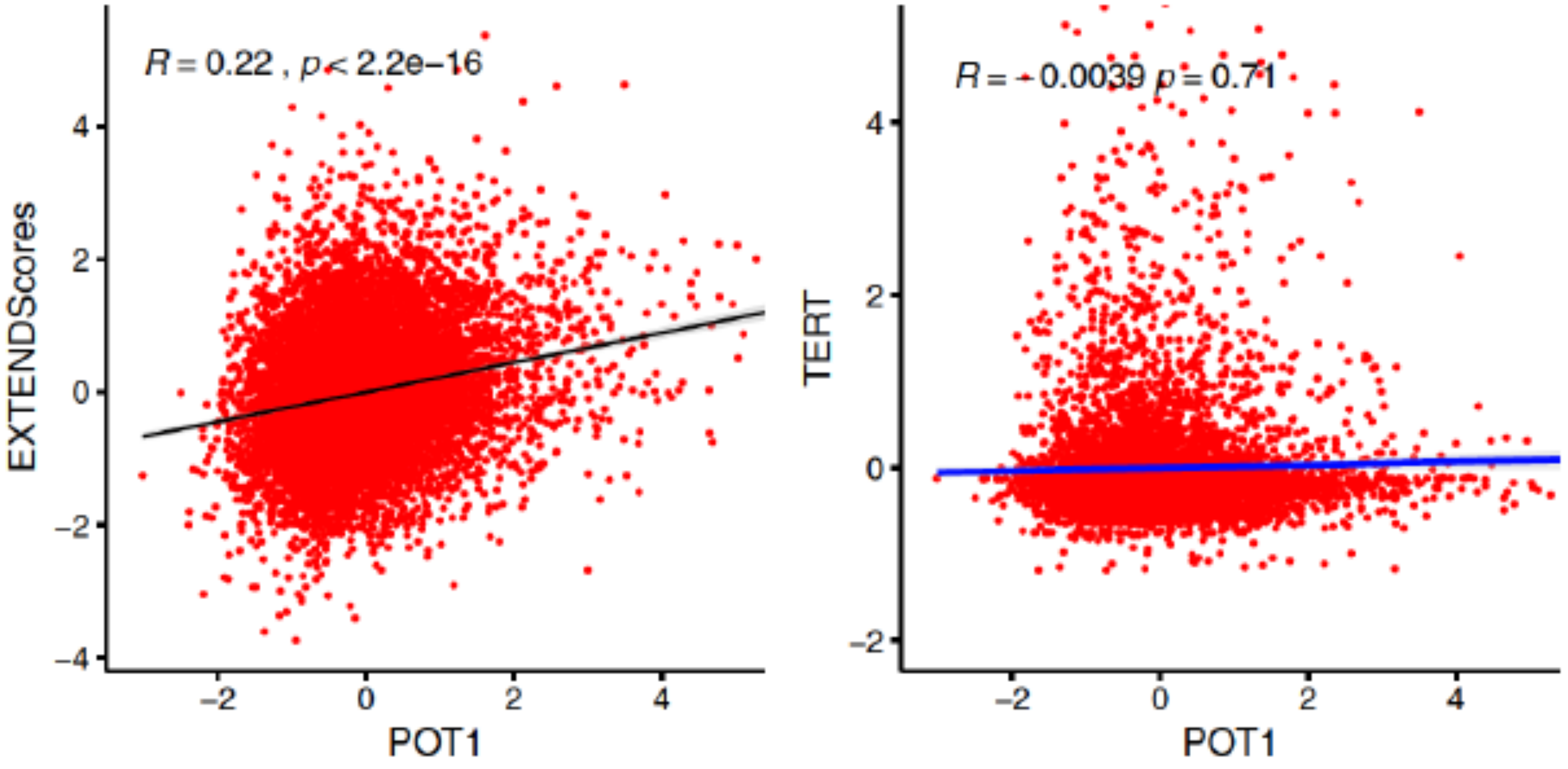

**Supplementary Fig. 14.** Correlation between telomere lengths (obtained from Barthel et al.,2017) and EXTEND scores across 31 cancer types (TCGA) for WGS and WXS platforms. Numbers in each cell (of heatmap) represent spearman correlation coefficient while the color (red = FDR < 0.05 and white = not significant; grey represents missing values)represents adjusted p value (multiple testing via Bonferroni correction).

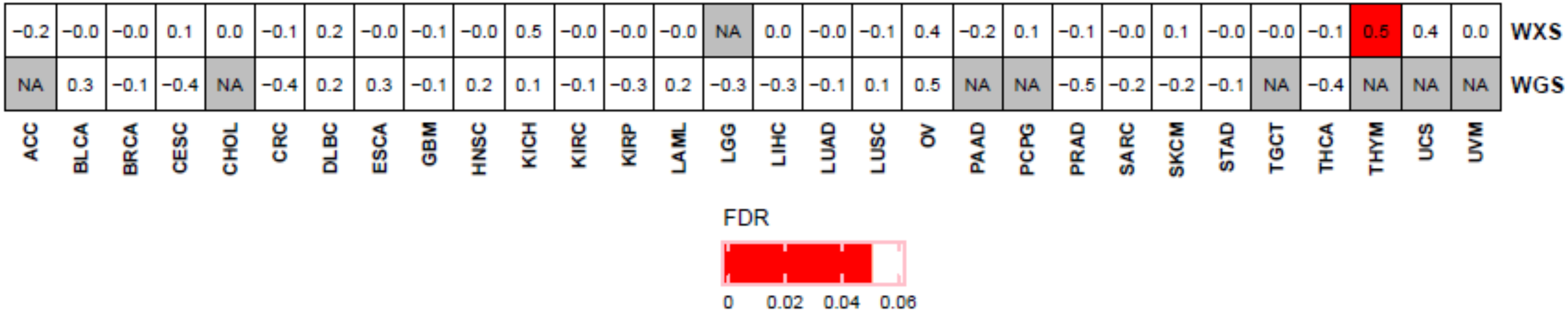

**Supplementary Fig. 15**. EXTEND scores across various subtypes in cancer cohorts (TCGA data). P-values were calculated using two-sided t-test. Boxplot interquartile ranges (25th to 75th percentile); middle bar defines median and the minima and maxima are within 1.5 times the interquartile range of the lower and higher quartile. Subtypes data used from TCGA.

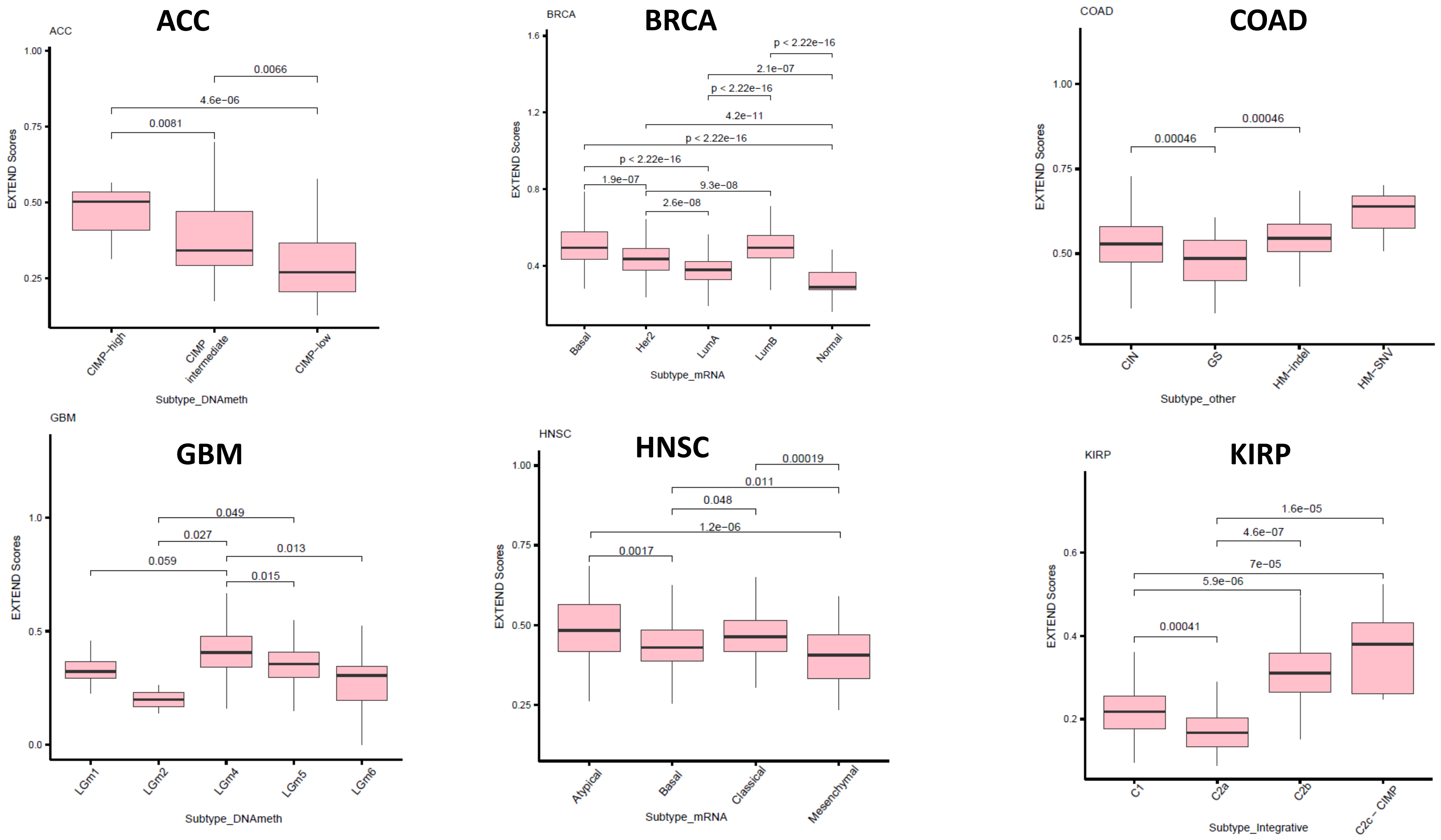

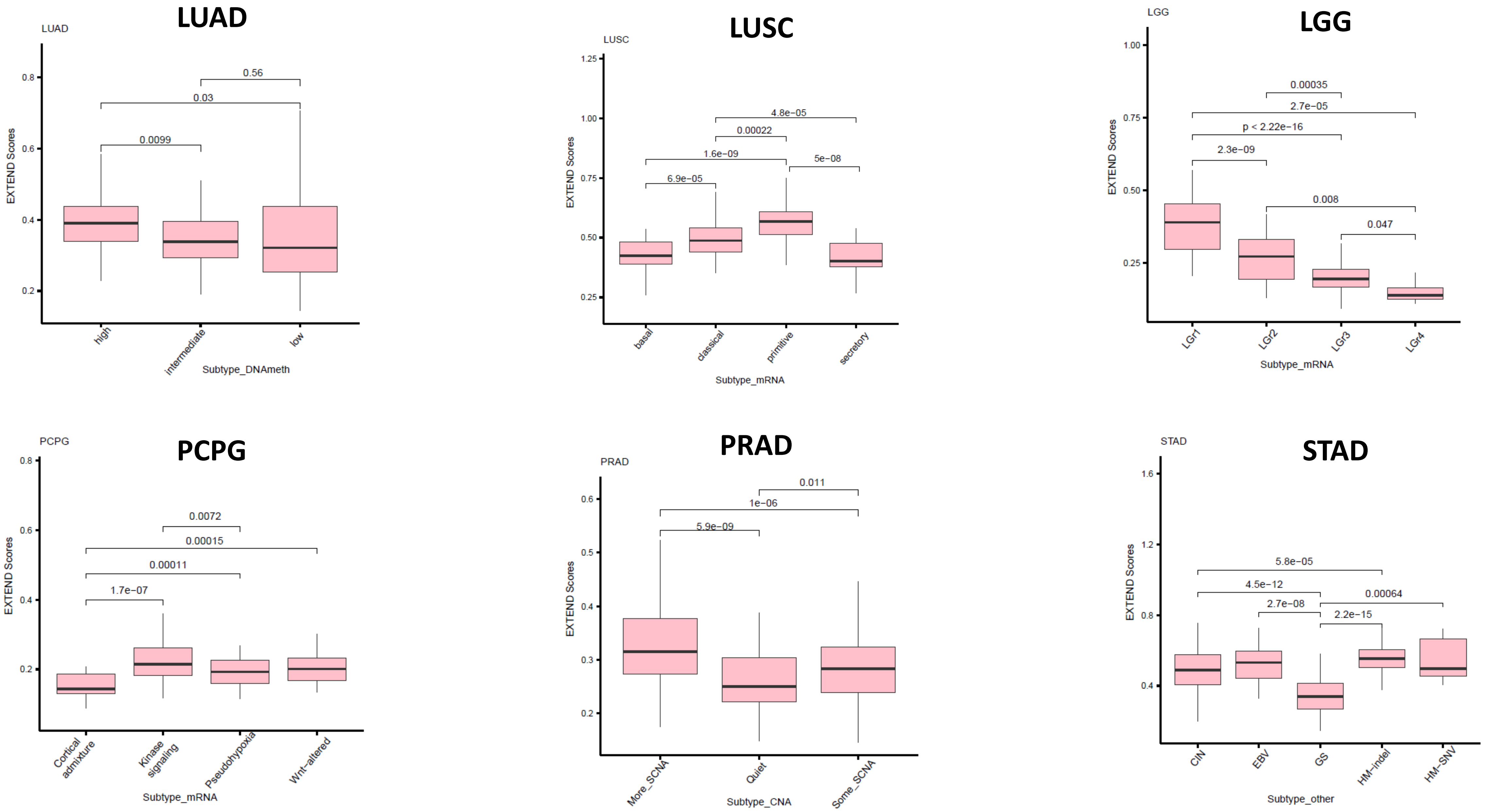

LUAD
LUSC
LGG
PCPG
PRAD
STAD
EXTEND Scores
Subtype_DNAmeth
high
intermediate
low
0.0099
0.03
0.56
Subtype_mRNA
basal
classical
primitive
secretory
6.9e-05
1.6e-09
5e-08
0.00022
4.8e-05
LGr1
LGr2
LGr3
LGr4
2.3e-09
p < 2.22e-16
2.7e-05
0.00035
0.008
0.047
Cortical admixture
Kinase signaling
Pseudohypoxia
Wnt-altered
1.7e-07
0.00011
0.00015
0.0072
Subtype_CNA
More_SCNA
Quiet
Some_SCNA
5.9e-09
1e-06
0.011
Subtype_other
CIN
EBV
GS
HM-indel
HM-SNV
5.8e-05
4.5e-12
2.7e-08
2.2e-15
0.00064

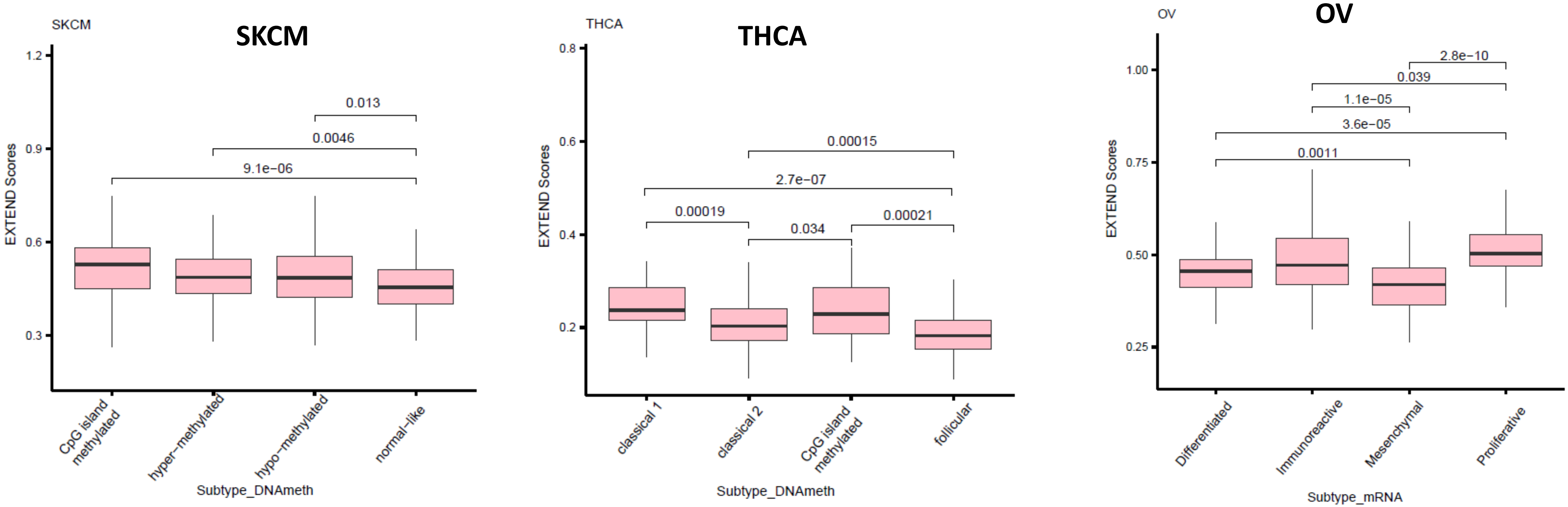
SKCM
SKCM
EXTEND Scores
1.2
0.9
0.6
0.3
0.013
0.0046
9.1e−06
CpG island methylated
hyper−methylated
hypo−methylated
normal−like
Subtype_DNAmeth
THCA
THCA
EXTEND Scores
0.8
0.6
0.4
0.2
0.00015
2.7e−07
0.00019
0.034
0.00021
classical 1
classical 2
CpG island methylated
follicular
Subtype_DNAmeth
OV
OV
EXTEND Scores
1.00
0.75
0.50
0.25
2.8e−10
0.039
1.1e−05
3.6e−05
0.0011
Differentiated
Immunoreactive
Mesenchymal
Proliferative
Subtype_mRNA

**Supplementary Fig. 16.** Correlation between telomerase activity and cancer stemness in medulloblastoma. **(a)** Scatter plot representing correlation (spearman rank method) of EXTEND scores with stemness index for single cell data (n = 5135 cells) from Medulloblastoma (Hovestadt et al., 2019). **(b)** Violin plot representing distribution of EXTEND scores across cycling cells (in G1-S and G2-M phase) and noncycling cells in Medulloblastoma data. P-values were calculated using two-sided t-test. Boxplot interquartile ranges (25$^{th}$ to 75$^{th}$ percentile); middle bar defines median and the minima and maxima are within 1.5 times the interquartile range of the lower and higher quartile. Data used is available in Source Data.

a

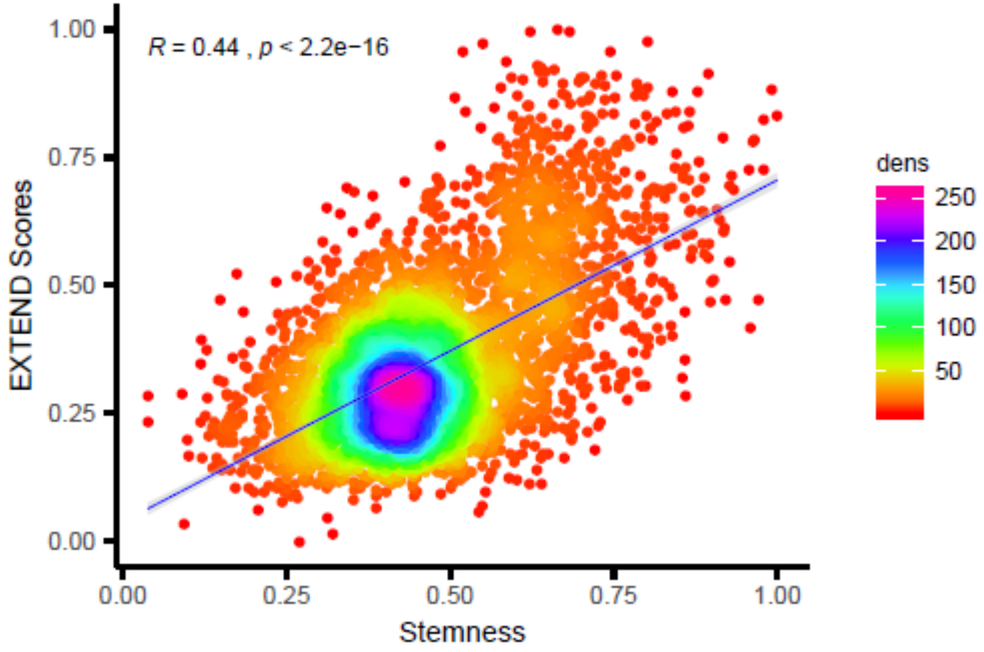


b

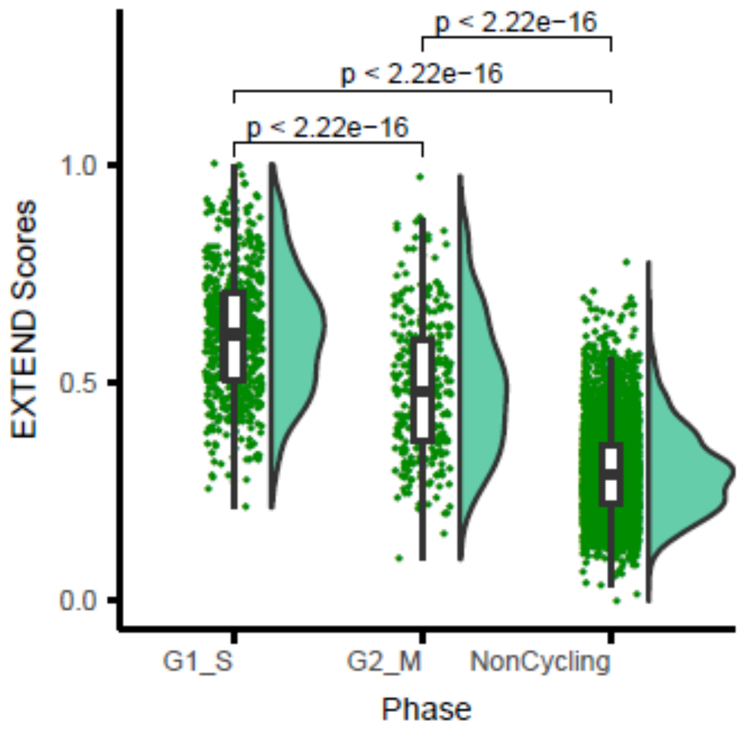

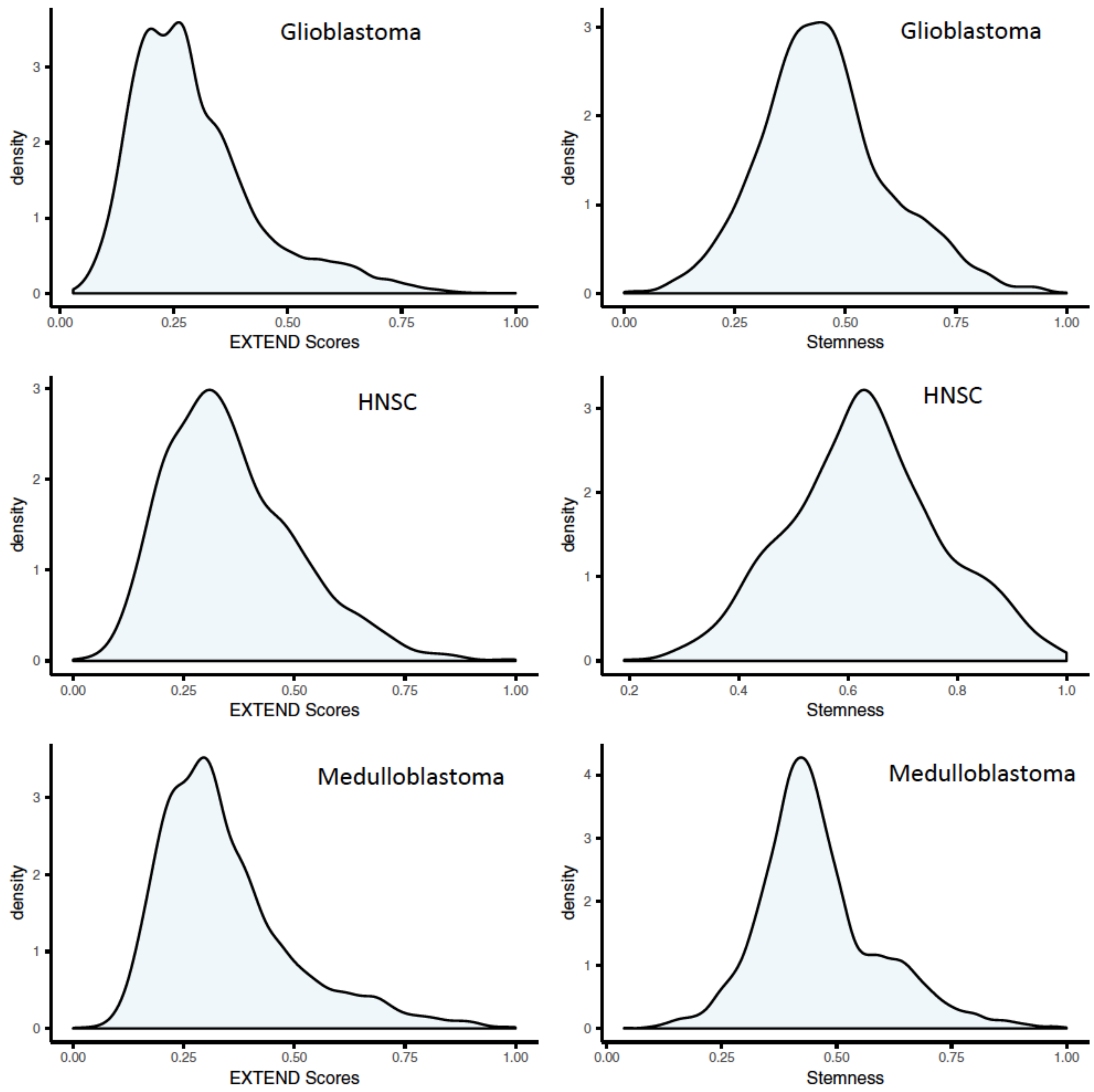


**Supplementary Fig. 17.** Distribution of EXTEND scores and cancer stemness scores. Their distributions consistently show multi-modes. Based on these distributions, we set EXTEND score to 0.5 as the cutoff to select for high stemness, high telomerase activity cells in the three single cell datasets (accounting for 10% of glioblastoma cells, 17% of head and neck cells, and 14% of medullo cells). Linear regression gives a slope of 0.67 for medulloblastoma, and 0.6 for both glioblastoma and head and neck cancer when considering EXTEND score as the dependent variable. Intercept is close to 0 for all three in linear regression. Data used is available in Source Data.
