# Supplementary material for: Integrated analysis of telomerase enzymatic activity unravels an association with cancer stemness and proliferation
Source: Nat Commun. 2021 Jan 8;12:139. doi: 10.1038/s41467-020-20474-9 (PMC7794223; doi:10.1038/s41467-020-20474-9)
Supplement: Supplementary file 3 — Description of Additional Supplementary Files [file 41467_2020_20474_MOESM3_ESM.pdf]

Description of Additional Supplementary Files

File Name: Supplementary Data 1
Description: EXTEND Signature Genes. Tissue expression pattern was obtained from GTEx data. The bottom half of the table lists details of the 108 TERT co-expressed genes in LGG, including correlation coefficient, p value, fold change in expression between TERTp mutant and ATRX altered tumors.

File Name: Supplementary Data 2
Description: Pathway enrichment of telomerase signature genes retrieved through MSigDB.

File Name: Supplementary Data 3
Description: EXTEND Scores for samples cross Genotype-Tissue Expression (GTEx) data

File Name: Supplementary Data 4
Description: EXTEND Scores for 31 cancer types in TCGA.

File Name: Supplementary Data 5
Description: EXTEND Scores and Stemness index correlation (Spearman rank test) based on permutations across TCGA cohort

File Name: Supplementary Data 6
Description: Pathways enrichment (MSigDB) for Up and downregulated genes based on high and low EXTEND Scores for 3 single cell data sets (Glioblastoma, Medulloblastoma and Head and Neck cancers).
