# Supplementary material for: Integrated analysis of telomerase enzymatic activity unravels an association with cancer stemness and proliferation
Source: Nat Commun. 2021 Jan 8;12:139. doi: 10.1038/s41467-020-20474-9 (PMC7794223; doi:10.1038/s41467-020-20474-9)
Supplement: Supplementary file 10 — Reporting Summary [file 41467_2020_20474_MOESM10_ESM.pdf]

Corresponding author(s): Siyuan Zheng

Last updated by author(s): Nov 30, 2020

Reporting Summary

Nature Research wishes to improve the reproducibility of the work that we publish. This form provides structure for consistency and transparency in reporting. For further information on Nature Research policies, see our Editorial Policies and the Editorial Policy Checklist.

Statistics

For all statistical analyses, confirm that the following items are present in the figure legend, table legend, main text, or Methods section.

n/a	Confirmed	
☐	☒	The exact sample size (*n*) for each experimental group/condition, given as a discrete number and unit of measurement
☐	☒	A statement on whether measurements were taken from distinct samples or whether the same sample was measured repeatedly
☐	☒	The statistical test(s) used AND whether they are one- or two-sided<br>*Only common tests should be described solely by name; describe more complex techniques in the Methods section.*
☐	☒	A description of all covariates tested
☐	☒	A description of any assumptions or corrections, such as tests of normality and adjustment for multiple comparisons
☐	☒	A full description of the statistical parameters including central tendency (e.g. means) or other basic estimates (e.g. regression coefficient) AND variation (e.g. standard deviation) or associated estimates of uncertainty (e.g. confidence intervals)
☐	☒	For null hypothesis testing, the test statistic (e.g. F, t, r) with confidence intervals, effect sizes, degrees of freedom and P value noted<br>*Give P values as exact values whenever suitable.*
☐	☒	For Bayesian analysis, information on the choice of priors and Markov chain Monte Carlo settings
☒	☐	For hierarchical and complex designs, identification of the appropriate level for tests and full reporting of outcomes
☐	☒	Estimates of effect sizes (e.g. Cohen's *d*, Pearson's *r*), indicating how they were calculated

Our web collection on statistics for biologists contains articles on many of the points above.

Software and code

Policy information about availability of computer code

Data collection	All genomic data sets were downloaded from public domains with versions and links cited in methods section of the manuscript. No software was used for data downloading.
Data analysis	Telomerase activity scores were generated using EXTEND package available at (https://github.com/NNoureen/EXTEND) while all other analysis codes are available at (https://github.com/NNoureen/EXTEND_datacodes). Software versions are Quantalife: Bio-Rad QX200; ComplexHeatmap: 2.20; R: 3.6.0.

For manuscripts utilizing custom algorithms or software that are central to the research but not yet described in published literature, software must be made available to editors and reviewers. We strongly encourage code deposition in a community repository (e.g. GitHub). See the Nature Research guidelines for submitting code & software for further information.

Data

Policy information about availability of data

All manuscripts must include a data availability statement. This statement should provide the following information, where applicable:

- Accession codes, unique identifiers, or web links for publicly available datasets
- A list of figures that have associated raw data
- A description of any restrictions on data availability

TCGA data were downloaded from Pancancer Atlas (https://gdc.cancer.gov/node/905/; synapse ID: syn4874822). CCLE expression data were downloaded from its website (https://portals.broadinstitute.org/ccle, CCLE_RNAseq_rsem_genes_tpm_20180929.txt.gz). GTEx data was downloaded from Genotype-Tissue Expression (GTEx) project portal (https://gtexportal.org/home/, v7). The following data were downloaded from GEO:
TERC manipulation dataset, GSE81507; Liposarcomas, GSE14533; Neuroblastomas, GSE120572; Medulloblastoma, GSE119926; Glioblastoma, GSE131928; HNSC, GSE103322;

Human development data set was downloaded from Array Express (E-MTAB-6814/).
Since all data are publicly available, no restrictions are applied to data availablity.

Field-specific reporting

Please select the one below that is the best fit for your research. If you are not sure, read the appropriate sections before making your selection.

- [x] Life sciences
- [ ] Behavioural & social sciences
- [ ] Ecological, evolutionary & environmental sciences

For a reference copy of the document with all sections, see nature.com/documents/nr-reporting-summary-flat.pdf

Life sciences study design

All studies must disclose on these points even when the disclosure is negative.

Sample size	All the available data was used in study
Data exclusions	Figure 3b: Only tumor types with a minimum of 10 cases for each stage were used in the analysis due to sample size consideration.
Replication	three sets of cell line experiment data were used to replicate the prediction accuracy of the algorithm.
Randomization	Random gene shuffling (permutation) was performed when evaluating correlation between telomerase and stemness. No other randomization was performed. Covariate was controlled in survival analysis using multivariate cox model.
Blinding	No blinding was necessary because phenotypes are expected to be associated with calculated scores.

Reporting for specific materials, systems and methods

We require information from authors about some types of materials, experimental systems and methods used in many studies. Here, indicate whether each material, system or method listed is relevant to your study. If you are not sure if a list item applies to your research, read the appropriate section before selecting a response.

Materials & experimental systems

n/a	Involved in the study	
[ ]	[x]	Antibodies
[ ]	[x]	Eukaryotic cell lines
[x]	[ ]	Palaeontology and archaeology
[x]	[ ]	Animals and other organisms
[x]	[ ]	Human research participants
[x]	[ ]	Clinical data
[x]	[ ]	Dual use research of concern

Methods

n/a	Involved in the study	
[x]	[ ]	ChIP-seq
[x]	[ ]	Flow cytometry
[x]	[ ]	MRI-based neuroimaging

Antibodies

Antibodies used	antibody against digoxigenin (anti-DIG-POD).
Validation	this antibody is part of the TeloTAGGG Telomerase PCR ELISA kit (Cat# 12013789001, Millipore Sigma). According to the vendor, the kit has been functionally validated.

Eukaryotic cell lines

Policy information about cell lines

Cell line source(s)	28 Glioma sphere forming cells from MD Anderson Cancer Center. Data for other cell lines used in this work were downloaded from previous publications and were cited in the manuscript.
Authentication	short tandem repeats using the Applied Biosystems AmpFISTR Identifier kit (Foster City, CA).
Mycoplasma contamination	not tested.
Commonly misidentified lines (See ICLAC register)	None.
